# Relationship between Barthel index with physical tests in adults with intellectual disabilities

**DOI:** 10.1186/2193-1801-3-543

**Published:** 2014-09-22

**Authors:** Antonio I Cuesta-Vargas, David Pérez-Cruzado

**Affiliations:** Departamento de Psiquiatría y Fisioterapia, Facultad de Ciencias de la Salud, Universidad de Málaga, Andalucia Tech, Cátedra de Fisioterapia y Discapacidad, Instituto de Biomedicina de Málaga (IBIMA), Grupo de Clinimetria (FE-14), Malaga, Spain; School of Clinical Science, Faculty of Health Science, Queensland University Technology, Brisbane, Australia

**Keywords:** Intellectual disability, Physical fitness test, Physical fitness, Physical condition, Barthel index, Relationships

## Abstract

We usually find low levels of fitness condition affect other aspects of living for people with ID like dependency in carrying out activivities of daily living. Therefore we find high levels of dependency in activities of daily living due to poor fitness condition. The aim of the study is to explore the criterion validity of the Barthel index with a physical fitness test. An observational cross-sectional study was conducted. Data from the Barthel index and a physical fitness test were measured in 122 adults with intellectual disability. The data were analysed to find out the relationship between four categories of the physical fitness test and the Barthel index. It needs to be stressed that the correlations between the Barthel index and leg, abdominal and arm strength can confirm that these physical test are predictive of the Barthel index. The correlations between the balance variables as functional reach and single-leg stance with eyes open shown relationships with Barthel Index. We found important correlations between the physical fitness test and the Barthel index, so we can affirm that some physical fitness features are predictor variables of the Barthel index.

## Background

There are studies that affirm that people with intellectual disability (ID) can’t do enough physical activity (PA) to obtain a beneficial effect for their health (Carmeli et al. 2002; Cuesta-Vargas et al. 2011; van Schijndel-Speet et al. 2014) because they found a lot of barriers to do physical activity (lack of social support and lack of clear policies for engaging in regular activity) (Bodde and Seo 2009; Mahy et al. 2010), so we know there is a lower physical fitness level in this population compared to the general population (Temple et al. 2006). PA provides many benefits to people with intellectual disability helping to reduce the risk of hypertension, coronary heart disease, stroke, diabetes, breast cancer and colon cancer, depression and falls, and is a key to energy balance and weight control (Takahashi et al. 2012). People with ID do not do enough physical activity so we find a reduction of their aerobic condition and neuromuscular profile (Wu et al. 2010). Physical activity is important for the increase of aspects of physical function, which allows the performance of more integrated functional tasks (Shields et al. 2013) such as muscle strength and power, balance, flexibility, or aerobic condition (Taylor et al. 2004).

We usually find low levels of fitness condition affect other aspects of living for people with ID, like exercising self-efficacy, and life satisfaction (Heller and Sorensen 2013), and includes dependency in carrying out activities of daily living (ADL). Therefore we find high levels of dependency in ADL due to poor fitness condition (Maring et al. 2013). Independency is the basic capacity of persons to care for themselves and to set common daily activities with higher levels of performance (Lin et al. 2013).

The Barthel Index (BI) is an ordinal scale used to measure performance in ADL (Cid-Ruzafa and Damián-Moreno 1997), this scale has been used in people with intellectual disabilities (Hilgenkamp et al. 2011; Chou et al. 2013; Maring et al. 2013) and similar scales have been used in other studies to measure dependency in people with ID (Carmeli et al. 2012), usually finding low values of dependency in ADL (Carmeli et al. 2012; Maring et al. 2013).

There is relation between a high level of dependency in ADL and mobility (Hilgenkamp et al. 2012) and in other studies they have found a significant relationship between a functional test and dependency in ADL (Maring et al. 2013).

The objective of this study is to know the relationship between Barthel Index with physical tests in adults with intellectual disabilities. Our hypothesis is that the physical fitness tests will have a moderate relationship with Barthel index.

## Materials and methods

### Design

This study is an observational cross-sectional study to discover the relationship between Barthel Index with physical fitness tests in adults with intellectual disabilities.

### Participants

The sample was 122 persons between 18 and 65 years old (37,96 ± 9,54) with ID (73 men and 48 women). The sample was recruited from the III National Special Olympics Championships (Madrid, Spain).

They had to be able to read and write in order to answer the questions and they had to be free of any disease that would prevent them from undertaking a physical activity. They have not any comorbid psychiatric or another disease.

Before starting the investigation we have guaranteed participants the protection of confidential information obtained from them [Law 15/1999 Protection of Personal Data]. Informed consent was obtained from all subjects, and study procedures were consistent with the Helsinki declaration.

### Procedure

Ten physiotherapists trained for this evaluation, two occupational therapists and one assistant measured the PFT and the BI.

All samples were evaluated with the BI and the PFT, both scales were explained to the patients and the scales were repeated if the participants did not understand; if a patient could not give us information about themselves, we asked a carer or a relative. We obtained demographic data of the sample too.

The PFT was explained repeatedly with the researcher showing how the test was done to facilitate understanding.

### Outcome measures

To measure physical condition we also reviewed the *Fun-fitness programme test*, Special Olympics (PFT); with this programme we could learn about the physical condition of the patients and help them with suggestions on how to improve their physical condition and to avoid injuries. When this test is performed, all participants receive information about their physical profile, and recommendations on how to increase their physical qualities (http://www.specialolympics.com). In our study we measured 12 items which cover strength, aerobic condition, balance and flexibility in the sample:

#### Passive knee extension (PKE)

The participant was positioned supine on a treatment table with hip and knee flexed at 90°. The passive knee extension was measured using a goniometer, with the fulcrum placed over the lateral femoral epicondyle and its arms in the direction of the greater trochanter and lateral malleolus respectively. Their ankle remained in a neutral position or in plantar flexion. If the knee went fully extended, the final value was recorded as 0°. If the knee did not extend, the value was recorded as negative (e.g., −40°). If the knee went beyond the fully straight position into hyperextension, the value was recorded as positive (e.g., +5°). The reliability of the PKE test was explored and compared with other clinical tests for assessing hamstring muscle as proposed by Gajdoski et al. (1993).

#### Calf Muscle Flexibility (CMF)

The participant was positioned supine on a table, with the hip and knee on the side to be measured in as much extension as possible. The fulcrum of the goniometer was placed over the lateral malleolus, with one of its arms in the direction of the fibular head and the other one in parallel to the lateral midline of the fifth metatarsal. Their ankle was passively dorsiflexed and its angle measured while their knee remained in extension. If the participant could not reach neutral position, the angle was recorded as negative (e.g., −10°). If the participant went beyond neutral, it was recorded as positive (e.g., +10°). If the participant only reached neutral, it was recorded as 0°. The reliability of this test can be found in Ekstrand et al. (1982).

#### Anterior Hip Flexibility (AHF)

The participant was positioned supine on a table, both hips flexed to 90°. The hip to be measured was flexed up to 100° with a hand beneath the lower back to ensure that it remained flattened. Opposite hip was kept at 90° and not allowed to move into extension during the test. The fulcrum of the goniometer was placed over the greater trochanter, with its arms aligned with the lateral midline of the pelvis and with the lateral midline of the femur respectively. The degrees of extension between the pelvis and thigh were measured before the pelvis began to move forward. If the thigh lowered to the table surface, the result was recorded as 0°. If the thigh did not reach the table, the angle was recorded as negative (e.g., −25°). The reliability of this test can be found in Ekstrand et al. (1982).

#### Functional shoulder rotation (FSR) (Apley’s Scratch Test)

The participant stood or was seated facing the back of a chair. The participant was instructed to reach one arm behind the head and down the back, while the other arm reached behind the hip and up the back. The participant was instructed to “try to touch their index fingers together.” A tape measure was used to measure the distance in cm between the index fingers in this position (one arm was in flexion/abduction/lateral rotation; the other was in extension/adduction/ medial rotation). The arm on top defined the recorded side (i.e., left arm on top = left; right arm on top = right). If the fingertips touched, the distance was recorded as 0. If the fingertips could not touch, the separation was recorded as negative (e.g., 15.2 cm). If the fingers overlap, the overlap was recorded as positive (e.g., +2.5 cm). The FSR is a reproducible measure of upper extremity function task that was validated in people with disabilities. The reliability of this test can be found in (Edwards et al. 2002; Boström et al. 1991).

#### The Timed-Stands Test (TST)

The timed-stands test was the method to quantify functional lower extremity muscle strength (hip and knee extension). The test requires the participant to complete 10 full stands from a seated position as quickly as possible without the use of their arms. The participant was seated in a firm straight-backed chair with the elbows flexed to 90° during the test. The participant had to stand 10 times as quickly as possible and the time to perform the task in minutes and seconds was recorded. If the participant could not perform 10 repetitions, the number of repetitions and the time taken was recorded. The TST is a reproducible measure of lower extremity function that was validated in people with disabilities. The reliability of this test can be found in Newcomer et al. (1993).

#### Partial Sit-Up Test (PSUT)

The partial sit-up test was the method to quantify abdominal muscle strength/endurance. The test requires the participant to complete as many sit-ups as possible from a supine position in one minute. The participant was positioned supine on a table or mat, with the legs placed on a chair or stool to keep their hips and knees bent at 90°. Their arms were placed straight out in front of the chest with the elbows extended during the entire test. Test-retest reliability and validity was established in a previous study (Faulkner et al. 1989).

#### Seated Push-Up (SPU)

The seated push-up test is a method of assessing the strength of the triceps, shoulder and scapular muscles. The test involves pushing the body up out of a seated position, and slowly lowering it back into the seat. The participant was placed with the knees out straight and the heels resting on the floor or table. The participant had to push their body up from the table or floor until the elbows were straight, held for 20 seconds and then slowly lowered back into the seat. The reliability of revised push-up test protocol in people without disabilities was 0.80 to 0.96 (Hong et al. 2011).

#### HandGrip Test (HGT)

The handgrip test is a standardized method for assessing strength of the hand and forearm muscles, as it has been correlated to upper extremity function. The test involved completing three grips on each side (preferred and non preferred hand) and recording the better of the three trials using an adjustable handgrip dynamometer. The participant had to keep the arm and hand at the side with the elbow bent at 90° while squeezing as forcefully as possible. The handgrip dynamometer have being found to be highly reliable (ICC = 0.98) and valid (ICC = 0.99) for measuring handgrip strength (Bellace et al. 2000).

#### Single-Leg Stance With Eyes Opened (SLSEO)

The single-leg stance test with eyes open is designed to assess balance with the assistance of visual cues. The test required the participant to stand on one leg with the eyes open. Balance must be maintained as long as possible. The arms were placed at the sides with elbows slightly flexed during the test. The test continued until participant lost balance, or put the other foot down (maximum time was 30 seconds). Interclass correlation coefficients were moderate to excellent (0.41 to 0.91) (Birmingham 2000).

#### Single-Leg Stance With Eyes Closed (SLSEC)

The single-leg stance test with eyes closed is similar to the previous one but without the assistance of visual cues, so the particpant’s eyes are kept closed or covered with a blindfold. Interclass correlation coefficients were moderate to excellent (0.41 to 0.91) suggesting that the standing balance tests are appropriate for distinguishing among group performances (Birmingham 2000).

#### Functional Reach Test (FRT)

The test requires the participant to reach forward beyond the length of his/her arm without loss of balance. The participant was on two legs, positioned shoulder width apart (or seated if the participant could not stand). The participant was requested to lift one arm up to 90°, forward flexion and extend fingers. Test-retest reliability and validity was established in a previous study (Duncan et al. 1990).

#### Two-minute step test (2MST)

Pre-exercise resting heart rate (RHR) was recorded with the participant seated before the test and again two-minute after the test is finished (2MAF). The participant was located next to a wall, and the minimum stepping height for the participant was marked. The test required a running tape measure from the iliac crest to the mid-patella, and to mark the midway point on the tape. This mark was transferred to the wall. The participant was requested to march for a maximum of two minutes, bringing each knee alternatively up to the tape mark in the wall. The number of times that the participant touched the tape with the right knee was recorded. The 2MST showed an acceptable reliability (0.63) (Burnstein et al. 2011; Brooks et al. 2002).

To measure the level of dependency we used the *Barthel index (Spanish version)*; this index provided us with quantitative information about the level of dependency, measuring the execution of ten daily life activities (Reliability of original version 0.86-0.92) (Cid-Ruzafa and Damián-Moreno 1997). Translated from the original version (Mahoney and Barthel 1965).

### Data analysis

We grouped the functional test into four categories: balance, flexibility, strength and aerobic condition for the better understanding and analysis of the data. The assignment was done in a similar way as Cuesta-Vargas (Cuesta-Vargas et al. 2013).

To check the homogeneity of the sample we carried out the Kolmogorov-Smirnov test to divide it into parametric and nonparametric measures. Mean and standard deviations of 95% confidence intervals of the values were calculated for each variable. A P-value < 0.05 was considered statistically significant.

We established a low correlation r ≤ 0.3; medium correlation r > 0.3 r ≤ 0.6 and high correlation r > 0.6 (Portney and Watkins 2009). Data was analysed using the SPSS package (version 19.0).

## Results

Descriptive results are presented in Table 1.

**Table 1:** **Characteristics of participants**

	Mean (standar deviation)
Size (m)	1,62 (0,12)
Weight (kg)	76,87 (15,30)
BMI	29,36 (5,05)
BI	94,11 (18,16)
FRT (cm)	33,90 (9,98)
SLSEO(s)	12,87 (10,7)
SLSEC(s)	3,94 (5,59)
PKE_R (°)	−22,14 (15,81)
PKE_L(°)	−22,96 (15,78)
CMF_R(°)	5,77 (9,79)
CMF_L(°)	5,28 (10,15)
AHF_R(°)	−5,95 (5,59)
AHF_L(°)	−6,57 (6,51)
FSR_R(cm)	−10,77 (12,82)
FRS_L(cm)	−13,24 (13,98)
TST(s)	22,05 (13,82)
PSUT(repetition/1 m)	28,18 (11,71)
SPU(s)	12,21 (8,47)
HGT(kg)	25,1267 (11,3)
2MEST_BE(bpm)	83,80 (13,53)
2MEST_AE(bpm)	121,02 (21,56)
2MEST_2MA(bpm)	87,47 (16,45)

N = 122.

BMI: Body mass index; FRT: Functional reach test; TUG: Timed up and go; SLSEO: Single-leg stance with opened eyes; SLSEC: Single-leg stance with closed eyes; PKE_R: Right passive knee extension; PKE_L: Left passive knee extension; CMF_R: Right calf muscle flexibility; CMF_L: Left calf muscle flexibility; AHF_R: Right anterior hip flexibility; AHF_L: Left anterior hip flexibility; FSR_R: Right functional shoulder rotation; FSR_L: Left functional shoulder rotation; TST: Time-stands test; PSUT: Partial sit-up test; SPU: Seated push-up; HGT: Handgrip test; 2MEST_BE: Two-minute step test_before exercise; 2MEST_AE: Two-minute step test_before exercise; 2MEST_2MA: Two-minute step test_2 minute after.

Correlations are presented in Table 2. We can see correlations of the physical fitness tests with the Barthel index. It needs to be stressed that the correlations between the Barthel index and leg strength (TST), abdominal (PSUT) and Seated Push-Up (SPU) are the highest relationship with Barthel index. Relationship with others physical fitness test are lower and not significant.

**Table 2:** **Correlations (Pearson r) between measures of dependency and physical fitness test**

	Balance			Flexibility							
	FRT	SLSEO	SLSEC	PKE_R	PKE_L	CMF_R	CMF_L	AHF_R	AHF_L	FSR_R	FSR_L
BI	,191*	,193*	,143	-,040	,071	-,095	-,045	-,143	-,032	,081	-,102
**Strenght**					**Aerobic condition**						
	**TST**	**PSUT**	**SPU**	**HGT**		**2MEST_BE**		**2MEST_AE**		**2MEST_2MA**	
BI	-,258**	,255**	,219*	,103		-,122		,052		-,087	

BI: Barthel index; FRT: Functional reach test; SLSEO: Single-leg stance with opened eyes; SLSEC: Single-leg stance with closed eyes; PKE_R: Right passive knee extension; PKE_L: Left passive knee extension; CMF_R: Right calf muscle flexibility; CMF_L: Left calf muscle flexibility; AHF_R: Right anterior hip flexibility; AHF_L: Left anterior hip flexibility; FSR_R: Right functional shoulder rotation; FSR_L: Left functional shoulder rotation; TST: Time-stands test; PSUT: Partial sit-up test; SPU: Seated push-up; HGT: Handgrip test; 2MEST_BE: Two-minute step test_before exercise; 2MEST_AE: Two-minute step test_before exercise; 2MEST_2MA: Two-minute step test_2 minute after.

*. r < 0,05.

**. r < 0,01.

## Discussion

This study was developed to know the relationship between the BI with PFT in people with intellectual disability. In the literature we found a study that found that correlations between balance and the BI are high in people with intellectual disability (r = 0.67) (Maring et al. 2013), but in this study the correlation is less (r ≈ 0.175). On the other hand, in adults with different disabilities the correlation between balance and the BI are less than in our study (Prata and Scheicher 2012). However, the most important is the results found by Maring (Maring et al. 2013) because the sample is similar to ours, although in this study they measured balance with the Tinetti Performance Oriented Mobility Assesment (POMA I) scale. The contrary happens if we analyse results in the flexibility categories.

Our study is the first that we know of that analyses the relationship between flexibility and level of dependency as we have not found any similar studies in the literature; with our results we can confirm that there is a low correlation between a functional test to measure flexibility and the BI.

On the other hand, contrary to flexibility, the results of the correlation between the BI and strength are similar to another study of people with intellectual disability, although in this study we find a higher correlation (Carmeli et al. 2012). This study is similar to ours because in this study they measured leg strength and hand strength with the Hand Grip test. There is a big difference between these studies in the results in the Hand Grip test. In our study the correlation with level of dependency is r = 0.103, but in Carmeli et al.’s study (Carmeli et al. 2012) r = 0.43; this could be due to the different measures of the level of dependency between the BI and the Katz index. Similar results are obtained if we compare the results of aerobic condition with other studies.

We did not find studies analysing the relationship between aerobic condition and the BI, but we can affirm that there is a low negative correlation that did not confirm the results of a similar study with people with intellectual disability (Carmeli et al. 2006). In this study, they used the BI to measure level of dependency in activities of daily living, but to measure aerobic condition they used SpO2%.

Four categories were formed using the PFT of FunFitness. These functional tests have been measured in other studies of people with ID, and we have confirmed that data obtained in the functional test in our study are similar to other similar studies (Cuesta-Vargas et al. 2011).

We can confirm data obtained with the BI to measure level of dependency too; in our study we found similar results to others that have measured levels of dependency with the BI (Maring et al. 2013; Hilgenkamp et al. 2011).

The strength of our study is the heterogeneous sample; our sample had a similar number of men and women and a large database because we have measured all functional tests that take a lot of time to measure.

The main weaknesses is that to our knowledge there are not many studies that use the same functional test to measure physical condition, so we were unable to compare our results better.

## Conclusions

The conclusion of our study is the knowledge of the relationship between PFT and BI, as we found a high correlation between 3 functional tests and BI: leg strength, triceps strength test and abdominal strength, so we can affirm that there are a mild relationship between level of dependency and strength. Other categories (flexibility, balance and aerobic condition) show low relationship with Barthel index.

## Abbreviations

ADL: Activities of daily living
ID: Intellectual disabilities
BI: Barthel index
PFT: Physical fitness test
PKE: Passive knee extension
CMF: Calf muscle flexibility
AHF: Anterior hip flexibility
FSR: Functional shoulder rotation
TST: Timed-stands test
PSUT: Partial sit-up test
SPU: Seated push-up
HGT: Handgrip test
SLSEO: Single-leg stance with eyes opened
SLSEC: Single-leg stance with eyes closed
FRT: Functional reach test
2MST: Two-minute step test.
